# Antimicrobial resistance—Do we share more than companionship with our dogs?

**DOI:** 10.1111/jam.15629

**Published:** 2022-05-29

**Authors:** Mari Røken, Kristin Forfang, Yngvild Wasteson, Anita Haug Haaland, Hans Geir Eiken, Snorre B. Hagen, Ane Mohn Bjelland

**Affiliations:** ^1^ Faculty of Veterinary Medicine, Department of Paraclinical Sciences Norwegian University of Life Sciences Ås Norway; ^2^ Division of Environment and Natural Resources Norwegian Institute of Bioeconomy Research Ås Norway; ^3^ Faculty of Veterinary Medicine, Department of Companion Animal Clinical Sciences Norwegian University of Life Sciences Ås Norway

**Keywords:** antimicrobial resistance genes, dog, faecal resistome, high throughput qPCR, human, one health

## Abstract

**Aims:**

To investigate and compare antimicrobial resistance genes (ARGs) in faeces from cohabiting dogs and owners.

**Methods and Results:**

DNA from faecal samples from 35 dogs and 35 owners was screened for the presence of 34 clinically relevant ARGs using high throughput qPCR. In total, 24 and 25 different ARGs were present in the dog and owner groups, respectively. The households had a mean of 9.9 ARGs present, with dogs and owners sharing on average 3.3 ARGs. ARGs were shared significantly more in households with dogs over 6 years old (3.5, interquartile range 2.75–5.0) than in households with younger dogs (2.5, interquartile range 2.0–3.0) (*p* = 0.02). Dogs possessed significantly more *mecA* and aminoglycoside resistance genes than owners.

**Conclusions:**

Dogs and owners can act as reservoirs for a broad range of ARGs belonging to several antimicrobial resistance classes. A modest proportion of the same resistance genes were present in both dogs and owners simultaneously, indicating that ARG transmission between the dog and human gut is of minor concern in the absence of antimicrobial selection.

**Significance and Impact of the Study:**

This study provides insight into the common dog and human gut resistomes, contributing to an improved knowledge base in risk assessments regarding ARG transmission between dogs and humans.

## INTRODUCTION

Antimicrobials are amongst the most prescribed medicines globally, and consumption continues to increase (Klein et al., 2018; Sriram et al., 2021). Bacteria have proven to be highly adaptive to antimicrobials, as they have managed to develop resistance mechanisms to nearly all antimicrobials shortly after they were introduced (Ventola, 2015). The use and misuse of antimicrobials in human and veterinary medicine have contributed to the global spread of drug‐resistant bacteria by driving the selection of bacteria in possession of antimicrobial resistance genes (ARGs) (Holmes et al., 2016). This complicates the treatment of infections in both human and veterinary medicine to such a degree that WHO has declared antimicrobial resistance one of the top 10 global public health threats to humanity (WHO, 2020a).

Humans live in an environment interacting with animals that may carry pathogens, which occasionally cross‐species barriers (WHO, 2020b). Animals are essential to humans both as a source of food and as companionship, but this relationship does not come without risks. The animal kingdom is a reservoir for micro‐organisms causing 60%–70% of infectious diseases in humans (Woolhouse & Gowtage‐Sequeria, 2005). Furthermore, most pathogens involved in emerging infectious disease events are caused by drug‐resistant strains (Jones et al., 2008). Companion animals are often in close direct contact with humans. For instance, dogs may share housing, food, sofas, and perhaps even beds with their owners. Hygienic measures like hand wash are not necessarily performed after direct or indirect contact with these animals. Hence, the potential for transmission of antimicrobial‐resistant (AMR) bacteria between companion animals and owners is present. As emphasized in an assessment report by the Norwegian Committee for Food and Environment, there is a lack of data regarding AMR reservoirs in pets and humans (VKM, 2015). This identified knowledge gap hampers the development of proper risk assessments.

Culturable AMR bacteria such as methicillin‐resistant *Staphylococcus* spp. (Ferreira et al., 2011) and extended‐spectrum β‐lactamase producing members of the Enterobacteriaceae family (Grönthal et al., 2018; Ljungquist et al., 2016) have received most of the attention as these are opportunistic pathogens and have been simultaneously isolated from cohabiting dogs and owners. However, non‐pathogenic gut commensals may also host ARGs (Bag et al., 2019). These bacteria may be overlooked since culture conditions for a significant part of the gut commensals are unknown (Juricova et al., 2021). To better understand the occurrence of ARGs, the possible interplay and exchange of ARGs between companion animals and their owners, and their respective gut resistomes must be explored more comprehensively and independently of isolation of specific bacterial species.

This study aimed to investigate and compare the presence of ARGs in faeces from cohabiting dogs and owners. Using high throughput qPCR, we screened faecal samples from 35 dogs and owners for the presence of 34 clinically relevant ARGs.

## MATERIALS AND METHODS

### Recruitment and enrolment criteria

This project was approved by the Regional Committee for Medical and Health Research Ethics Southeast, approval number: 62346. Participants were recruited and samples were collected through the HUNT4‐One Health survey (NTNU, 2019; NMBU, 2020). All participants signed consent forms before enrolment. A total of 836 dogs participated in the survey. Questionnaires about the dogs' breed, health condition, diet, activities and primary use were sent to the owners after sample collection. One hundred and eleven completed questionnaires were returned. Dog and owner pairs (*n* = 35) were selected from the pool of 111 dogs based on the following criteria: The dog's primary use was being a family dog, and the owner considered the dog's health condition to be good or excellent at the time of sampling. To avoid the formation of subgroups amongst the dogs, sledge dogs, hunting dogs and dogs who underwent antimicrobial treatment or had gastrointestinal symptoms at the time of sampling were excluded. No information on antimicrobial use was available through the HUNT study for the owners. However, all the owners participating in this study had submitted self‐evaluation scores of their health with answering options poor, not so good, good and excellent. In addition, participants had reported whether they suffered from any long‐standing illness or injury of a physical or psychological nature impairing their function in their daily lives with answering options yes or no.

### Sampling

All participants received written instructions and a video link on how to collect faecal samples. Participants collected about a teaspoon of fresh faeces using EasySampler for stool collection (GP Medical Devices), gloves, and a wooden spatula to apply faeces on a collection card (LipiDx). The same participants collected faecal samples from their respective dogs and applied them to collection cards. The collection cards were left to dry for approximately 2 h and then put into separate sterile envelopes. Samples were sent to the HUNT Biobank by mail for storage at −20°C until further handling and genomic DNA extraction.

### DNA extraction

Depending on the visible amount of faecal material on the collection card, one to two 8 mm biopsy punches from the dog samples (*n* = 35) were used for DNA extraction. One 6 mm biopsy punch from the human collection cards was used for the analysis. One 8 mm punch from empty collection cards was included as a negative control for each extraction batch (*n* = 4). The DNA extractions were performed using the QIAamp PowerFaecal Pro DNA kit (Qiagen GmbH) according to the manufacturer's protocol. For the bead beating step, we used the TissueLyser II system at 30 Hz for 10 min. We used the supplied C6 solution as elution buffer with a final volume of 50 μl. Quantification of eluted DNA was performed by Qubit 3.0 fluorometer using dsDNA Broad Range Assay Kit (Invitrogen). The DNA quality was measured using a NanoDrop™ ND‐1000 spectrophotometer (Thermo Scientific). The eluates were stored at 4°C for no more than 4 days before 20 μl were sent overnight on ice for HT‐qPCR analysis.

### HT‐qPCR analysis

The qPCR analysis was performed at the Norwegian Institute of Bioeconomy Research (NIBIO) using a high‐throughput setup with the Biomark HD system for real‐time PCR (Fluidigm). Pre‐amplification was performed with 1.25 μl of DNA and a final primer concentration of 0.05 μmol l^−1^ in a 14‐cycled specific target amplification. The primers used are listed in Table 1. The pre‐amplification conditions were as follows: Initial denaturation at 95°C for 15 min, 14 cycles at 95°C for 15 s and 60°C for 4 min. The presence of ARGs in the faecal samples was determined using a qPCR chip with 46 assays developed to detect 34 ARGs. We selected these ARGs based on the list of indicators by Berendonk et al. (2015) and expanded with other clinically relevant ARGs. The ARGs are responsible for genotypic resistance to 10 antimicrobial classes, including beta‐lactams, tetracyclines, aminoglycosides, amphenicols, fluoroquinolones, sulphonamides, dihydrofolate reductase (DHFR) inhibitors, glycopeptides, colistin, and macrolide‐lincosamide‐streptogramin B (MLS). In addition, the chip contained two assays for the detection of microbial DNA (16S rRNA) and the class 1 integron‐integrase gene (*intl1)*. Eleven positive controls with confirmed presence of specific ARGs and four negative controls were included in each run. The chip was primed and loaded with pre‐amplified DNA (2.25 μl) and EvaGreen assays (Invitrogen) in two replicates according to the manufacturer's protocol. Initially, the samples were thermal mixed at 70°C for 40 minutes, followed by 60°C for 30 s. Then, the thermal profile was: Initial hot start at 98°C for 2 min, 40 cycles at 98°C for 5 s, and 60°C for 20 s, ending with a melting curve analysis at 60°C for 3 s followed by a 1°C/3 s increase to 95°C. All 46 assays were tested against standard curves of the 11 positive controls to determine the slopes and intercept for quantification of each assay. Data collection was performed using Biomark HD Data Collection software (Fluidigm, USA). The positive controls were used to correct the cycle threshold (CT) value before quantification. Quantification of ARGs present was conducted in Fluidigm Real‐time PCR analysis software (version 4.5.2) using Equation 1, in which the CT value represented the mean of the duplicates.

(1) $$\mathrm{ARG}\left(\frac{\mathrm{ng}}{\mathrm{\mu l}}\right)=10\left(\frac{\mathrm{CT}+{\mathrm{CT}}_{\text{corr}}-\text{intercept}}{\text{slope}}\right)$$

**TABLE 1:** List of primers included in the qPCR chip for detection of ARGs

Assay	Forward primer	Reverse primer	References
16S_1	CCCAGATGGGATTAGCTTGT	TCTGGACCGTGTCTCAGTTC	Kim and Lee (2014)
aac6_1	CTGTTCAATGATCCCGAGGT	TGGCGTGTTTGAACCATGTA	Hu et al. (2013a, 2013b)
aac3_2	GCGCACCCCGATGCMTCSATGG	GGCAACGGCCTCGGCGTARTGSA	Heuer et al. (2002)
ant3_1	CAGCGCAATGACATTCTTGC	GTCGGCAGCGACAYCCTTCG	Walsh et al. (2011)
ant3_2	ATCTTGCGATTTTGCTGACC	TGTACCAAATGCGAGCAAGA	Szczepanowski et al. (2009)
aph3_2	ATTCAACGGGAAACGTCTTG	ACGCTACCTTTGCCATGTTT	Szczepanowski et al. (2009)
blaACT_3	GTRCCGGATGAGGTCRMGGAT	TGGYRTTRGCGTAAAGACG	Chavda et al. (2016)
blaCTX_2	GCGATAACGTGGCGATGAAT	GTCGAGACGGAACGTTTCGT	Zhu et al. (2013)
blaCTX_3	CGTCACGCTGTTGTTAGGAA	CGCTCATCAGCACGATAAAG	Szczepanowski et al. (2009)
blaDHA_1	AACTTTCACAGGTGTGCTGGGT	GCTGCCACTGCTGATAGAA	Pérez‐Pérez and Hanson Nancy (2002)
blaKPC_1	GGCAGTCGGAGACAAAACC	CCCTCGAGCGCGAGTCTA	Chen et al. (2012)
blaNDM_1	TTGGCGATCTGGTTTTCC	GGTTGATCTCCTGCTTGA	Zheng et al. (2013)
blaNDM_2	CGCAACACAGCCTGACTTT	TCGATCCCAACGGTGATATT	Ong et al. (2011)
blaSHV_1	TCCCATGATGAGCACCTTTAAA	TCCTGCTGGCGATAGTGGAT	Roschanski et al. (2014)
blaTEM_1	GCATCTTACGGATGGCATGA	GTCCTCCGATCGTTGTCAGAA	Roschanski et al. (2014)
blaVIM_1	GGTCTCATTGTCCGTGATGGTGATGAG	CTCGATGAGAGTCCTTCTAGAG	Kaczmarek et al. (2006)
blaVIM_2	TGGCAACGTACGCATCACC	CGCAGCACCGGGATAGAA	Weiß et al. (2017)
blaVIM_3	GCACTTCTCGCGGAGATTG	CGACGGTGATGCGTACGTT	Zhu et al. (2013)
catA_2	GGGTGAGTTTCACCAGTTTTGATT	CACCTTGTCGCCTTGCGTATA	Zhu et al. (2013)
cmlA_3	TAGTTGGCGGTACTCCCTTG	GAATTGTGCTCGCTGTCGTA	Szczepanowski et al. (2009)
dfrA_2	GAGCTGAGATATACACTCTGGCACT	GTACGGAATTACAGCTTGAATGGT	Grape et al. (2007)
ermB_1	GGTTGCTCTTGCACACTCAAG	CAGTTGACGATATTCTCGATTG	Koike et al. (2010)
ermB_2	GGATTCTACAAGCGTACCTTGGA	TGGCAGCTTAAGCAATTGCT	Schmidt et al. (2015)
ermB_3	GGATTCTACAAGCGTACCTTGGA	AATCGAGACTTGAGTGTGCAAGAG	Belén Flórez et al. (2014)
ermF_1	TCGTTTTACGGGTCAGCACTT	CAACCAAAGCTGTGTCGTTT	Schmidt et al. (2015)
ermF_2	TGATGCCCGAAATGTTCAAGT	AAAGGAAATTTCGGAACTGCAA	Belén Flórez et al. (2014)
floR_2	ATTGTCTTCACGGTGTCCGTTA	CCGCGATGTCGTCGAACT	Zhu et al. (2013)
intl1_1	CCTCCCGCACGATGATC	TCCACGCATCGTCAGGC	Bass et al. (1999)
mcr1_2	ACACTTATGGCACGGTCTATG	GCACACCCAAACCAATGATAC	Bontron et al. (2016)
mecA_1	CATTGATCGCAACGTTCAATTT	TGGTCTTTCTGCATTCCTGGA	Francois et al. (2003)
oqxA_3	GCGATGATGCTCTCCTTTCT	GATCGACTTCACCAGCACCT	Pitt et al. (2020)
oqxB_1	TCCTGATCTCCATTAACGCCCA	ACCGGAACCCATCTCGATGC	Kim Hong et al. (2009)
qnrA1_1	ATTTCTCACGCCAGGATTTG	CAGATCGGCATAGCTGAAG	Marti and Balcázar (2013)
qnrB1_2	GGMATHGAAATTCGCCACTG	TTYGCBGYYCGCCAGTCG	Cattoir et al. (2007)
qnrS_1	GACGTGCTAACTTGCGTGAT	TGGCATTGTTGGAAACTTG	Marti and Balcázar (2013)
strA_3	CCAGTTCTCTTCGGCGTTAG	ACTCTTCAATGCACGGGTCT	Faldynova et al. (2013)
strB_2	CGGTCGTGAGAACAATCTGA	ATGATGCAGATCGCCATGTA	Pyatov et al. (2017)
sul1_3	ACGAGATTGTGCGGTTCTTC	CCGACTTCAGCTTTTGAAGG	Li et al. (2007)
sul2_2	CTCCGATGGAGGCCGGTAT	GGGAATGCCATCTGCCTTGA	Luo et al. (2010)
sul3_3	TTCGTTCAGCGAATTGGTGCAG	TTCGTTCACGCTTTACACCAGC	Muziasari et al. (2014)
tetA_3	CTCACCAGCCTGACCTCGAT	CACGTTGTTATAGAAGCCGCATAG	Zhu et al. (2013)
tetB_2	GCCCAGTGCTGTTGTTGTCAT	TGAAAGCAAACGGCCTAAATACA	Zhu et al. (2013)
tetM_2	TAATATTGGAGTTTTAGCTCATGTTGATG	CCTCTCTGACGTTCTAAAAGCGTATTAT	Zhu et al. (2013)
vanA_1	CTGTGAGGTCGGTTGTGCG	TTTGGTCCACCTCGCCA	Volkmann et al. (2004)
vanA_2	AGCTGTACTCTCGCCGGATA	CGCAGCCTACAAAAGGGATA	Cantarelli et al. (2011)
vanA_3	GCCGGAAAAAGGCTCTGAA	TTTTTTGCCGTTTCCTGTATCC	He et al. (2020)

### Statistical analysis

Statistical analysis was performed using JMP® Pro Software (Version 15.2.1, SAS Institute Inc.). The quantitative output of ARGs was transformed to binominal values and treated in general as categorical variables in the statistical analysis. Fisher's exact test was applied when comparing the presence/absence of genes between dogs and owners at the group level. When comparing the number of ARGs and antimicrobial resistance classes between the groups, the data were treated as continuous variables, and a two‐tailed Mann–Whitney test was applied. The significance level was set at 5%. The mean values are reported with their corresponding interquartile ranges (IQR). Pearson's correlation coefficient was used to assess the correlation between the number of ARGs in dogs and owners at the household level.

## RESULTS

### Enrolment and participant data

Of the 35 dogs enrolled, 24 were purebreds from 19 different breeds, and 11 dogs were of mixed or unknown breeds. The group consisted of 17 males and 18 females, four of whom were neutered. Their median age was 6 years. Twelve dogs had never received antimicrobial treatment; eight dogs had received antimicrobial treatment between one and three times. Three dogs had received antibiotics more than three times. Four owners did not recall whether their dogs had been treated with antibiotics during their lifetime.

The owner group consisted of 18 women and 17 men with a median age of 55 years. Of these, 29 considered their health to be good or excellent. None of the participants reported their health to be poor, whilst five considered their health not good. Twenty‐three owners reported not to be suffering from any longstanding illness or injury of a physical or psychological nature impairing their functioning in their daily lives, whilst 12 reported suffering from this.

### Analysis

Of the dog and owner samples, 69/70 tested positive for the presence of microbial DNA (16S rRNA). The negative sample was of canine origin and was excluded from further analysis. The owner of this dog was included in the analysis of human samples but excluded from the household level analysis, making the number of participating households 34. Three negative batch controls tested positive for low amounts of 16S rRNA, including one testing positive for the *ant(3′)* gene. Due to suspicion that the *ant(3′)* positive control had been contaminated during the first qPCR run, it was rerun under the same conditions. The control was then negative for *ant(3′)*; however, positive for low concentrations of the 16S rRNA gene.

### Antimicrobial resistance genes

**FIGURE 1:**
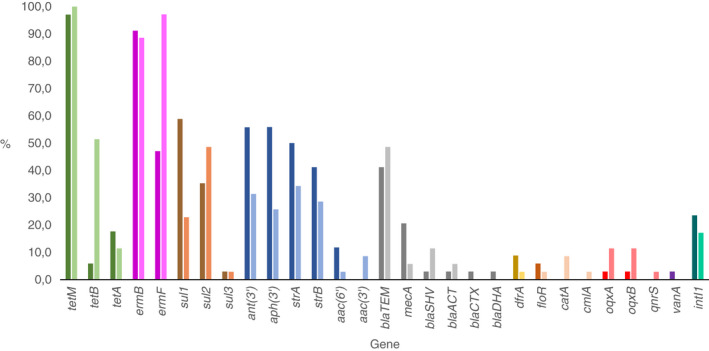
Detected antimicrobial resistance genes and the class 1 integron‐integrase gene intI1 in dogs, represented by the darker shades, and owners, represented by lighter shades. Different colours represent the different antimicrobial classes. From left to right: Tetracyclines, macrolides‐lincosamides‐streptogramins (MLS), sulphonamides, aminoglycosides, beta‐lactams, dihydrofolate reductase (DHFR) inhibitors, amphenicols, fluoroquinolones, glycopeptides, and the class 1 integron‐integrase gene intI1.

Our results show that 68/69 dog and owner samples tested positive for two or more ARGs. The remaining sample was of canine origin and lacked all the targeted ARGs. The detected ARGs in dogs and owners are listed in Figure 1 and Table 2. Overall, 28 different ARGs were detected in the human and canine samples combined, 24 ARGs in dogs and 25 ARGs in humans. The mean number of ARGs was 6.7 (IQR: 4.0–9.25) amongst the dogs and 6.7 (IQR: 4.‐10.0) amongst the owners. The most frequently occurring ARGs in the dog group were *tetM* (97.1%, 33/34), *ermB* (91.2%, 31/34), *sul1* (58.8% 20/34), and *ant(3′*) (58.8%, 20/34). Likewise, *tetM* was the most frequent ARG amongst the owners, detected in all (100%, 35/35) samples, followed by *ermF* (97.1%, 34/35) and *ermB* (88.6%, 31/35). Seven dogs (20.6%) and two owners (5.4%) tested positive for the *mecA* gene. None of the dog nor owner samples tested positive for *qnrA1*, *qnrB1*, *mcr1*, *bla*_KPC_, *bla*_NDM_ or *bla*_VIM_.

**TABLE 2:** Results of the detection of antimicrobial resistance genes and their corresponding antimicrobial classes in dogs and owners. Numbers represent the percentage of individuals testing positive and the percentage of households in which both dog and owner tested positive for the same ARG. Listed *p*‐values refer to differences in gene occurrence between dogs and owners. The class 1 integron‐integrase gene intI1 is included at the bottom of the table. DHFR = dihydrofolate reductase. MLS = macrolide‐ lincosamide‐streptogramin B.

Antimicrobial resistance class	Antimicrobial resistance gene	Dogs %	Owners %	*p*‐value	Households with shared gene %
Aminoglycosides	*aac(6′)*	11.8	2.9	0.1981	0.0
	*aac(3′)*	0.0	8.6	0.2391	0.0
	*ant(3′)*	58.8	31.4	**0.0301**	14.7
	*aph(3′)*	55.9	25.7	**0.0146**	17.6
	*strA*	50.0	34.3	0.2270	11.8
	*strB*	41.2	28.6	0.3185	5.9
Amphenicols	*catA*	0.0	8.6	0.2391	0.0
	*cmlA*	0.0	2.9	1.0000	0.0
	*floR*	5.9	2.9	0.6139	0.0
Beta‐lactams	*bla*_ACT_	2.9	5.7	1.0000	0.0
	*bla*_CTX_	2.9	0.0	0.4928	0.0
	*bla*_DHA_	2.9	0.0	0.4928	0.0
	*bla*_KPC_	0.0	0.0	—	0.0
	*bla*_NDM_	0.0	0.0	—	0.0
	*bla*_SHV_	2.9	11.4	0.3565	0.0
	*bla*_TEM_	41.2	48.6	0.6307	23.5
	*bla*_VIM_	0.0	0.0	—	0.0
	*mecA*	20.6	5.7	0.0840	2.9
Colistin	*mcr1*	0	0	—	0
DHFR inhibitors	*dfrA*	8.8	2.9	0.3565	0
Glycopeptides	*vanA*	2.9	0	0.4928	0
MLS	*ermB*	91.2	88.6	1.0000	79.4
	*ermF*	47.1	97.1	<**0.0001**	47.1
Quinolones	*oqxA*	2.9	11,4	0.3565	0.0
	*oqxB*	2.9	11,4	0.3565	0.0
	*qnrA1*	0.0	0.0	—	0.0
	*qnrB1*	0.0	0.0	—	0.0
	*qnrS*	0.0	2.9	1.0000	0.0
Sulphonamides	*sul1*	58.8	22.9	**0.0033**	5.9
	*sul2*	35.3	48.6	0.3319	23.5
	*sul3*	2.9	2.9	1.0000	0.0
Tetracyclines	*tetA*	17.6	11.4	0.5130	0.0
	*tetB*	5.9	51.4	**<0.0001**	2.9
	*tetM*	97.1	100	0.4928	97.1
Class 1 integron‐integrase	*intI1*	23.5	17.1	0.5613	8.8

Significant *p*‐values are emphasized in bold.

Of the ARGs analysed, 61.8% (21/34) were equally represented in the two groups. The remaining 38.2% (13/34) ARGs were unique to one, or their presence differed significantly between the groups. Four of the ARGs, *floR*, *bla*_CTX_, *bla*_DHA_ and *vanA*, were unique to the dog group. The *aac(3′)*, *catA*, *cmlA* and *qnr*S genes were found exclusively amongst the owners. Five ARGs, *ermF*, *tetB*, *ant(3′)*, *aph(3′)* and *sul1*, occurred in both groups but with significantly different frequencies (Table 2). The *ermF* gene was detected in 97.1% (34/35) of the owner samples and 47.1% (16/34) of the dog samples. Worth noticing is that 81.2% (13/16) of the *ermF*‐positive dogs were at the median age of six or older, making it the only gene associated with age (*p* = 0.0342). In general, dogs possessed a wider range of aminoglycoside resistance genes than the owners (Table S1); 64.7% (22/34) of the dogs tested positive for two or more aminoglycoside resistance genes, compared to 37.1% (13/35) of the owners (*p* = 0.0306). Concurrent carriage of *ant(3′)* and *aph(3′)* occurred in 38.2% (13/34) of the dogs, compared to 11.4% (4/35) of the owners (*p* = 0.0125). In addition, 10 of these dogs tested positive for *strA* and *strB*, thus contributing to the high number of aminoglycoside resistance genes.

### Class 1 integron‐integrase gene (int *I* 1)

**FIGURE 2:**
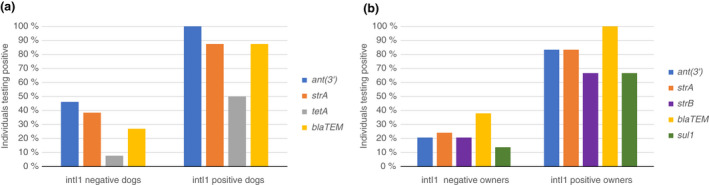
Distribution of selected ARGs in intI1 positive and negative dogs (a) and owners (b).

Eight dogs (23.5%) and six owners (17.1%) tested positive for the *intI1* gene. The mean number of ARGs detected in the *intI1‐*positive dogs was 9.4 (IQR: 7.25–12.75), significantly higher than the *intI1* negative dogs' mean of 5.9 (IQR: 4.0–7.5, *p* = 0.0257). The difference relied on more *intI1* positive dogs possessing *ant(3′)* (*p* = 0.0109), *strA* (*p* = 0.0391), *bla*_*TEM*_ (*p* = 0.0039) and *tetA* (*p* = 0.018) compared to the *intI1* negative dogs (Figure 2). We observed the same association amongst the *intI1* positive owners with a mean of 10.5 ARGs (IQR: 9.25–11.5) compared to *intI1* negative owners with a mean of 5.9 ARGs (IQR: 4.0–7.5, *p* = 0.0009). The *intI1‐*positive owner samples contained more *ant(3′)* (*p* = 0.0047), *bla*_*TEM*_ (*p* = 0.006), *strA* (*p* = 0.0082), *strB* (*p* = 0.0477) and *sul1* (*p* = 0.0096) compared to the samples of the *intI1* negatives.

### Household‐level

**FIGURE 3:**
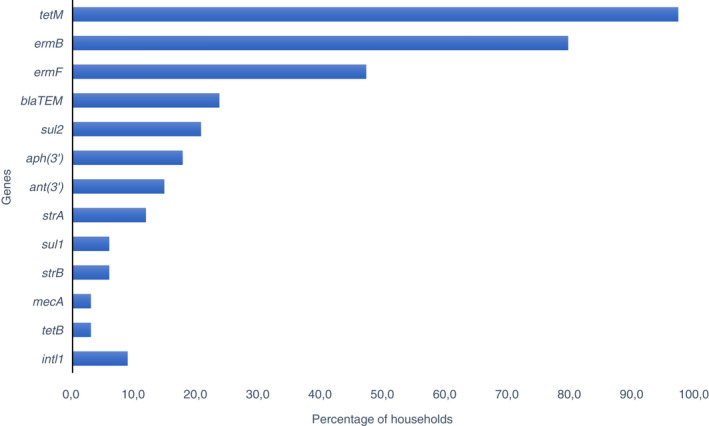
Percentage of households in which dogs and owners possessed the same ARGs. tetM and ermB were the dominating shared ARGs in the 34 households tested.

**FIGURE 4:**
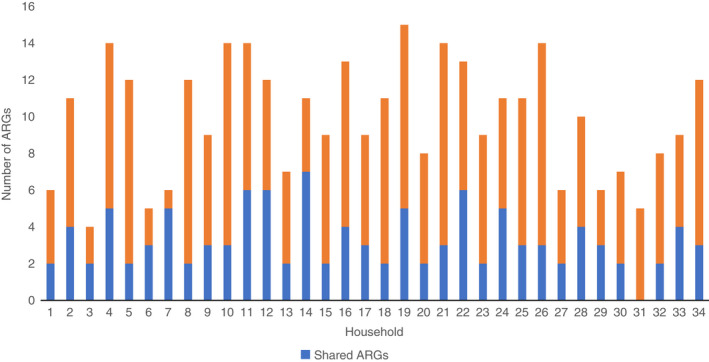
Total number of unique ARGs detected in the different households presented as columns. The blue proportions of the columns present the number of shared ARGs in the households. The total number of unique detected ARGs ranged between 4 and 15 in the households, whilst the shared ARGs ranged between 0 and 7.

On average, we detected 9.9 (IQR: 7.0–12.25) different ARGs in each of the 34 households included in the study. In total, 35.3% (12/34) of the different ARGs were identified simultaneously in both dogs and owners. These genes confer genotypic resistance to aminoglycosides, beta‐lactams, MLS, sulphonamides and tetracyclines (Figure 3). We observed close to no correlation between the number of ARGs detected in cohabiting dogs and owners (r [32] = −0.11 *p* = 0.52). On average, dogs and owners had 3.3 (IQR: 2.0–4.25) ARGs in common. All except one household had a minimum of two shared ARGs, the exception being the household in which the dog tested negative for all ARGs (Figure 4). Households with dogs aged 6 years and older shared significantly more ARGs (3.5, IQR: 2.75–5.0) than households with younger dogs (2.5, IQR: 2.0–3.0) (*p* = 0.0204). The difference relied mainly on *ermF* being shared in 59% (13/22) of the older‐dog households versus 18.2% (2/11) of the younger‐dog households (*p* = 0.0342). Furthermore, in seven older dog households, both dog and owner had positive matches on *sul2*, whilst none in the younger dog households shared this gene. However, this difference was not significant (*p* = 0.0674). For one household, the dog's age was not listed and was excluded from the analysis. The *intI1* gene was simultaneously present in the dog and owner in three cases (Table S1). These dog‐owner pairs had two, four and seven ARGs in common, respectively.

## DISCUSSION

Literature on the occurrence of common ARGs amongst cohabiting dogs and humans is scarce, and few studies, e.g. Kim et al. (2020) and Liu et al. (2021), have focused on the canine gut resistome. Therefore, we aimed to describe the canine resistome and investigate to what degree cohabiting dogs and owners share ARGs in the gut by screening the samples for a panel of 34 ARGs and the class 1 integron‐integrase gene *intI1*. Although most of the investigated ARGs were equally represented in both groups, the dogs and owners had few ARGs in common (3.3 ARGs on average) at the household level.

Our results show that tetracycline and MLS resistance genes were the most abundant ARGs irrespective of host species. These results correspond well with previous research on human faecal samples (Feng et al., 2018; Hu et al., 2013a, 2013b; Seville et al., 2009) and seem to comply with the dog samples as well. In striking contrast to our results that show a high representation of *ermB* in the dogs, Kim et al. (2020) did not detect any *ermB* genes amongst the canine faecal samples they investigated. Similar to us, Kim et al. (2020) found the tetracycline‐ and MLS resistance genes to be the most occurrent ARGs.

The slight majority of ARGs were equally present in both groups. However, 38.6% of the ARGs were unique to one group, or their presence differed significantly. The limited sample size may have contributed to the ARGs being unique to one group or absent in all samples. Nevertheless, the differences in the prevalence of *sul1* and *tetB* between dogs and owners may point to species‐specific compositional differences between the canine and the human gut microbiome. The *ant(3′)* gene was significantly more occurrent in the dog samples. Concurrent carriage of *aph(3′)* and, in many cases, also *strA* and *strB* contributed to a higher total number of aminoglycoside resistance genes amongst the dogs. According to the NORM‐VET surveillance programme, the usage of aminoglycosides is low in Norway (NORM, 2020). In faecal samples from healthy dogs, the surveillance programme reports a low aminoglycoside resistance level in *Escherichia coli*, *Enterococcus faecium* and *Enterococcus faecalis*. Hence, bacteria hosting the aminoglycoside resistance genes detected in the dog samples were most likely other bacteria. Our findings emphasize the importance of maintaining the low usage of aminoglycosides in small animal clinical practice to avoid the selection and dissemination of aminoglycoside‐resistant bacteria.

Surprisingly many of the dog samples tested positive for *mecA*, *the gene mediating* methicillin resistance in staphylococci. The *mecA* gene is often associated with *Staphylococcus pseudintermedius* in dogs. However, it may also be present in coagulase‐negative staphylococci (MRCoNS) and *Staphylococcus aureus* (MRSA), the latter being more often associated with humans (Gómez‐Sanz et al., 2019; Turner et al., 2019; Weese & van Duijkeren, 2010). A prevalence screening of methicillin‐resistant *S. pseudintermedius* (MRSP) in healthy dogs in Norway showed carriage rates of 2.6% (5/189) (Kjellman et al., 2015). Additionally, the 2019 surveillance report on antimicrobial resistance in Norway stated that none out of 230 healthy dogs carried methicillin‐resistant staphylococci, whilst 4.5% (7/157) of the *S. pseudintermedius* clinical isolates were identified as MRSP (NORM, 2020). Staphylococci are primarily associated with skin and mucosal membranes (Bannoehr & Guardabassi, 2012; Foster, 2002). Our results may partly reflect the self‐contamination of the faeces from these sites and not the state in the gut. Still, the level of *mecA* positive samples was notably high considering the low reported prevalence of methicillin‐resistant staphylococci in Norwegian dogs. The HT‐qPCR method used in this study may have contributed to the high number of *mecA‐*positive individuals, as it can detect low‐abundance genes (Franklin et al., 2021; Waseem et al., 2019) and does not discriminate between different staphylococcal species. Hence, the *mecA* may originate from other sources such as coagulase‐negative staphylococci that frequently carry *mecA (*Garza‐González et al., 2010 *)*.

In this study, individuals carrying *intI1*‐positive bacteria had more ARGs in the gut than individuals who were negative for *intI1*. We expected this as the *intI1* gene encodes the integrase in class 1 integrons, enabling the integrons to capture and express a wide range of resistance genes (Lacotte et al., 2017). Class 1 integrons can be carried by conjugative plasmids and are thus believed to be a significant contributor to the acquisition and dissemination of ARGs (Gillings et al., 2017). However, a study by Zhang et al. (2018) suggested that the contribution of class 1 integrons to the dissemination of ARGs might be limited as they are mainly within *Gammaproteobacteria*. Furthermore, Zhang et al. showed that more than half of the class 1 integrons were chromosomally embedded with less potential for horizontal gene transfer. In this study, eight dogs and six owners tested positive for *intI1*, of which three dog‐owner pairs simultaneously carried the gene. Seeing that class 1 integrons are considered almost universal in the microbiota of humans and domesticated animals (Gillings, 2017), the number of *intI1* carrying individuals in this study was notably low. Moreover, the low number indicates a limited transmission rate of *intI1‐*carrying bacteria between dogs and owners.

Considering the close contact humans and their pets often have, it is surprising that dogs and owners from the same household had such a small proportion of the same ARGs in common. Undoubtedly, factors such as species barriers, the extent of contact in the individual homes, and the limited sample size may have affected the results. The observed association between shared ARGs and age may imply that the dogs' age and perhaps even cohabiting time are factors that affect the degree of common ARGs. Whether this is caused by the inter‐species transmission of bacteria, a shift in the dogs' microbiomes with age, or is purely coincidental, remains unanswered. Resistance determinants persist for at least a year in the human gut (Forslund et al., 2013). With that in mind, our results suggest that the exchange of ARGs between dogs and owners and subsequent carriage of ARGs are of limited concern. However, the situation might have looked differently if the dog or owner had undergone antimicrobial treatment. In which case, the selection pressure would increase the population of resistant bacteria and potentially increase the risk of exposure to either the dog or owner (Francino, 2016).

The HT‐qPCR approach used in this study proved to be a quick and efficient method to screen for multiple ARGs in many samples simultaneously. The technique is often used to detect ARGs in environmental samples as it requires a limited amount of DNA per sample and can detect low abundance genes (Franklin et al., 2021; Waseem et al., 2019). Nevertheless, some studies have successfully applied the method to detect ARGs in faecal samples from animals and humans (Zhao et al., 2018; Zhou et al., 2018). A downside of the method is that it fails to connect the ARGs to the host bacteria. However, the method's strength is that it enabled us to identify ARGs from the whole faecal microbiome, not only ARGs in culturable faecal bacteria. As exemplified in this study, low‐biomass samples, like negative controls are prone to contamination as DNA is ubiquitous and can even be found in DNA extraction kits (Karstens et al., 2019; Saladié et al., 2020; Salter et al., 2014). Therefore, we accepted that some of the controls contained low amounts of the 16S rRNA gene*. We suspected that the ant(3′)‐*positive negative control had been contaminated by a neighbouring well due to the close positioning of the wells. A targeted rerun of this specific sample confirmed this assumption. The pre‐amplification step of the method improves the detection limit but may also reduce the specificity of the analysis leading to false positives (Sandberg et al., 2018). A metagenomic sequencing analysis may be another option, as it provides data on the taxonomic composition of the gut microbiome as well as detecting ARGs. However, detecting low‐abundance genes requires high‐depth sequencing, which may be challenging and costly to achieve (Waseem et al., 2019).

In conclusion, despite a reported low level of antimicrobial resistance in Norway (NORM, 2018, 2019, 2020), a wide range of ARGs belonging to several AMR classes was present in faecal samples from both dogs and owners. Thus, both groups may act as reservoirs for bacteria carrying these ARGs. A modest proportion of the same resistance genes was present in both dogs and owners simultaneously. This indicates that the transmission of resistance genes between dogs and owners is of limited concern, provided a low antimicrobial selection pressure. Furthermore, this study has provided valuable insight into the common dog and human resistome and improved the knowledge base for risk assessments regarding the zoonotic potential of antimicrobial resistance.

## CONFLICT OF INTEREST

No conflict of interest was declared.

## Supporting information


Table S1
